# Editorial Department

**Published:** 1882-07

**Authors:** 


### The Bistoury.

ELMIRA, N. Y., JULY, 1882.

The BISTOURY is published Quarterly, upon the first of April, July, October and January, at Fifty Cents a year in advance.

### Editorial Department.

EDUCATIONAL.—Read Dr. Blackham's contribution, entitled "Blind Man's Buff." There is a deal in it worth reflecting over.

WOODSY.—You will find but little in this department of the journal, this time, that has not a flavor of the woods. Indeed, it might—in imitation of some magazines of more pretention—have inscribed across its face, "THE CAMPING NUMBER."

TWO WEEKS LATE.—Our subscribers have learned, by this time, the cause of delay in issuing the July number of THE BISTOURY. The editor—as usual for him at this season of the year—"went a-fishing," and did not prepare his "copy" before he went.

AMATEUR PHOTOGRAPHY.—There were two photographers in the Bodines camping party, this year, who made it exceedingly lively for the rest of their companions. The ladies of the expedition were photographed "within an inch of their lives." Every attitude they assumed—that was picturesque—was immediately seized upon by these camera men as a subject for a picture. They were pictured at table—in hammocks—in boats on the pond. Stood in groups upon the rocks—amid the daisies—in the green fields—on the farm wagon—in every conceivable place and position did these ladies pose for the indomitable and persevering picture men. Let a bevy of them get together, for a pleasant chat, under the shade of some picturesque tree, when at once they would hear the command—"there! hold still a moment—that's a beautiful picture—be quiet now—one, two, three! that will do!" and the picture is made. Those two picture chaps made one hundred and fifty negatives each—there is a woman in every view, and all of them still living.

THOSE DOCTORS' BILLS.—Dr. Bliss is in great danger of having his bill for his enforced services upon the President so curtailed as to leave small reward for his bold and reckless conduct in that sad affair. At this distance, it looks as though no pay at all would more than compensate him for the part he played in that tragedy. It is bad enough for a respectable physician to crowd out and keep out the regularly recognized physician to a case, without soliciting pay for the nefarious practice. We hope Congress will spare the nation every *unnecessary* expense in this matter, and so give Dr. Bliss his just and proper dues.

AMERICAN SOCIETY OF MICROSCOPISTS.—The annual meeting of this society of scientists is announced to occur in Elmira, commencing August 15th and continuing four days. The meetings will be held in the Park Church, in the auditorium of which President Blackham's address will be given on the evening of the first day. Two sessions a day will be held, commencing at 9:30 a. m. and 2:30 p. m. On the evening of the 17th, a soiree will be given by the National Society to the citizens of Elmira, at which a fine display of microscopical instruments and objects will be shown. On the 18th, the Elmira Microscopical Society will tender the American Society an excursion and banquet. The meeting will be an interesting and profitable one to scientific men, and will attract hither distinguished men in science from all parts of the United States. Every effort is being made by the Elmira Society to give their visitors a pleasant time, in which undertaking they will be heartly aided by all citizens.

A KICK.—Did you ever see a cow kick? Perhaps that question does not appear in the least interesting at the first glimpse. But, *did* you? When a horse kicks, he does it methodically, and aims his blow at the object upon which he means to exhibit his hatred. A mule, it is said, can hit a quarter of a dollar, twice the length of his body, with either hind hoof. But a cow—goodness! Why, a cow can hit a tin cup anywhere within a radius of ten feet, at one single and unprovoked kick. We tried to analyze that kick, but it could not be done. W—, the photographer of the expedition—attempted instantaneous views of it, while we tickled her with a straw. The cameras, stationed at the four points of the compass, were all demolished, in the experiment, and the plates "fogged" before we could reclaim the fragments from the ruins. We know a Philadelphia photographer that will give a handsome premium for an instantaneous group of the kick of a cow—and no questions asked.

---

DON'T GRUNT.—One of the most disagreeable habits that can possibly be cultivated is that of continually canvassing your ailments—"grunting." "How did you rest last night?" "O, very poorly. I turned over in bed a hundred times, and just couldn't sleep, and my back ached so—." O, pshaw! What is the use in making everybody miserable with the recital of your discomforts? Keep them to yourself. Nobody is benefited by hearing of your sickness, real or imaginary; nor does relief or mitigation come to yourself. We knew an old chap who invariably replied to the salutation of "How are you this morning?" "O, not well at all!" yet would eat fourteen buckwheat cakes and a pound of sausage for breakfast. He was a chronic grunter, and never knew when he *did* feel well.

Another miserable habit is that of really telling one how you do feel, when you are asked the question as a mere matter of form or salutation. We have been detained twenty minutes to hear a fellow tell his symptoms, when we simply saluted him with "How are you?" Then, we have seen other gamey fellows, who had sickness and suffering written all over their features, reply "Splendid!" to the same salutation, when both of us knew "there was a lie out." That is the sort of pluckiness that wins one's respect and admiration. A heroic liar—one that practices the art for the benefit of his fellows, deserves our applause.

We have observed still another trait of character that is grotesque because of the great confidence exhibited in the physician and his medicines. There be those who, for every trivial discomfort of body, expect an immediate remedy. A slight headache, "stitch in the side," twinge of pain in the stomach, sleepless night, or if they wink fourteen times in a minute, when the average number of gyrations of the eye-lids should be but twelve, a medicine, a specific, is expected and demanded at once. Such people—and they be numerous—are chronic grunts. They not only make themselves miserable by watching and attaching importance to every trivial matter of this sort, but are a downright nuisance to those with whom they are daily thrown in contact. Stop your grunting. Keep your little discomforts to yourself. Say you feel gloriously when you don't. Lie about it, and you will soon deceive yourself, even if you fail in the attempt upon your friends.

---

THAT NEW CODE.—Since a regularly appointed committee of distinguished physicians of the New York State Medical Society recommended a revision of the code of ethics that *permits* regular physicians consulting with homœopaths, there has been a great commotion created among the members of the American Medical Society, who, in their recent meeting at St. Paul, denounced in unmeasured and unselected terms the action of the State society.

Gentlemen, keep cool. If all the success in combating disease belonged to any special body of men, or any one school of medicine, and all the remaining ones were failures and blunderers, it might possibly do to exhibit your wrath and displeasure in being *permitted*—permitted, mind you—to consult with all legally qualified physicians (in times of great emergency), if you choose. We see nothing very terrible in all this. If we did not "choose" to consult with them, why we would not, and we cannot see anything in the code that will compel one to.

Since the practice of medicine is entirely empirical, and since one "school" seems to have equal success with the others in not killing their patients, no one of them have any right to proclaim infallibility over the others. The test of a good physician should be the extent of his scientific acquirements and his success in combating disease. It matters little to the patient employing him whether he accomplishes the cure through the administration of large or small doses of medicines, or no medicine at all, if only it is done. Then, if anything is to be learned from consultations with homœopathic physicians—and their success certainly equals ours in treating disease, measuring it by the recoveries made by their patients—by all means let us have it. If nothing is learned, certainly nothing is detracted from whatever skill we may possess, beside giving us an opportunity of imparting a modicum of it to our benighted brother. Gentlemen of the medical profession, look at this question as you will, no tenable excuse can be found for not consulting with any respectable gentleman of any school of medicine when a life is at stake. No harm, no disgrace can come to you from such a consultation. Give your advice everywhere, when solicited, for the benefit of the suffering, and let ethics and societies take care of themselves.

---

HAPPENINGS IN CAMP.—The yearly excursion to Bodines-on-the-Lycoming was made this season commencing June 19th and continuing to July 15th. The party was made up of Hamlin, a Scranton editor, Charles the philosopher, a Philadelphia photographer, five ladies, three children, Thad, Jr., Way, Fritz, the editor of this journal, and William, the cook, in all, sixteen people. Six large tents afforded shelter for the party, and two boats did service upon the pond. We started in a drenching rain, but arrived at the grounds under a clear sky.

The first three days were occupied in making camp, putting up tents, constructing paths, building foot-bridges, arranging wild flower beds and otherwise beautifying our surroundings. Everything being made ready for comfortable living, we gave ourselves up to the enjoyments to be gleaned from camp life. The first night of our stay in the woods was made memorable by the visitation of some large animal that banqueted upon a ham which we had suspended from a beech tree. We examined the ground for tracks of the ferocious beast—having visions of bear or catamount—but none could be clearly made out. We therefore, set a steel trap at the foot of the tree the following night, thinking we might capture the beast. In the morning the trap was gone. Then, surely it was surmised that a bear had invaded our grounds and made off with ham and trap. While speculating upon the affair at breakfast that morning, Squire Bodine appeared from the neighboring farm-house, and holding our steel trap at arms length delivered this message: "See here, you chaps must be more keerful how you set your traps around here. I found this on my dog's hind foot this mornin'!" And so ended our vision of wild beasts and the mystery of the ham theft.

The Editor and Doctor of the party went up stream one pleasant morning to build a raft of flood-wood that the camp-fire might be the better suplied, and with which to construct tables, benches and other household furniture. The ride down the rapids resulted in complete wreck of the craft, lodging the mariners high (but not dry) on a rock in the middle of the stream. They were rescued without aid of breeches buoy or surf boat, while their raft was safely harbored—in fragments—by the other campers at the camp below. Another beautiful morning found the photographers, with Hamlin and Charles on their way to the famed slope wall, there to fish and take views of the pretty scenes in that locality. They there encountered a minister who delights much in pursuing the gentle art, and who is quite noted for his skill in casting the fly. After a half hour pleasant chat with him, while all were seated upon a log under the shadow of a wide-spreading beech, one of the photographers took a rod from his companion's hand and shortly hooked a magnificent fish. He ran the rapids with him, shouting and screaming every time the fish left the water. The campers were equally exultant, following the lucky fisherman, offering all sorts of advice as to the landing of the prize, until it was safely brought to net. During the excitement not one word of satisfaction escaped the lips of our ministerial friend, not even a smile was noticed upon his face, nothing was done or said by him that naturally would be expected from an old fisherman under such circumstances. He stood solemnly by a pool, casting his fly mechanically, and never deigned to look in the direction of the happy throng engaged in landing the large trout. Thereat we all wondered, and commented on our way to camp upon his stolidity. That evening about the camp-fire we were enlightened as to the utter indifference manifested by the dominie at the landing of that magnificent trout. The 'squire, with whom the minister was stopping, let the secret out somewhat in the following style: "Well, you're a pretty lot of fellows, arn't you?"

"What's up now, squire?"

"Why, the dominie pricked a nice trout up by the slope wall, last night, and went up on purpose, this mornin', to ketch him. He found you chaps there, sat down on a log to wait for you to go, and blamed if one of you didn't go and ketch his trout, right under his nose!"

A shout greeted this announcement, and at once explained the preacher's indifference, or perhaps disgust, at the capture and landing of his fish.

In a photographic excursion to Astonville—a little, deserted mining village, four miles above our camp—we were besieged on every hand by the fair country damsels to have us take their pictures. They all wanted "tin types," at ten cents each, which they declared to be the regulation price with itinerant picture-men, like ourselves. While the two artists made photographs of the old furnaces and the ruins round about, Charles flirted with and told stories to the damsels, much to their entertainment and delight. He promised them all a picture, so that when we returned to him, he poised them on the briar-covered stumps, and on the fences; grouped them fantastically in the hot and dusty road; had them march and counter-march before him while he peered at them through the camera, and then had them sit, perfectly motionless, for an hour-and-a half, while he took an "instantaneous" picture of them. When next we passed that way they importuned him for the pictures. He told them they were all "under-exposed"—time of sitting not long enough—would call again and have them sit an hour longer, when he hoped he would succeed better.

As we walked along the mountain road, we noticed that the woodchucks and other animals were warned of our approach by the alarm call of the crows. These ever-watchful and sly birds were feeding their squalling, chattering young wherever we went. The old ones always gave a certain note of alarm when we came near. Hearing this, the woodchucks would sit erect and peer over the clover tops to see the direction of our approach. Squirrels, we noticed, would scamper for cover when this signal of danger was given them.

Reaching camp one day, the ladies and children had much to say of a deer that swam down the pond, and within the length of a fishing pole of camp. William, the cook, pursued him in a boat, thinking to dispatch him with an ax, but the nimble creature soon eluded him and disappeared up the mountain side. On the following day a large catamount clambered down a log into the creek, to drink, when, seeing the campers, he crooked his back, gave a blood-curdling wail, and left much more expeditiously than he came. His cry was heard that same night upon the mountain, tending to disturb the quiet and repose of the women and children.

In a month sojourn in the woods, as indeed elsewhere, one may expect some rainy days. They came to us, and, although not absolutely unendurable, they were most annoying if not distressing to one camper, who never before had slept in a tent. He arrived in a rain that continued uninterruptedly for two days and nights. He wrapped himself in all the available blankets and shawls, incased his feet in rubber boots, and elevated them upon camp stools, hoisted his umbrella and indulged himself in imaginary chills and attacks of neuralgia, rheumatism and gout. As he sat toasting his back by the camp-fire, with drooping eyelids and solemn visage, he presented a picture of despair. At the end of the second day, he declared his intention of going home, asserting his belief that the sun had never shone, nor would it, in the valley of the Lycoming, and that a trout had never been seen, much less captured, in its muddy waters. He constantly extracted from his luggage and intently examined thermometers, barometers and hygrometers, invariably returning them to their cases with a sigh and multifold expressions of condemnation of the weather prospects. Charles continually chaffed him upon his array of instruments, declaring that he carried with him everything but the "holy bible, square and compass." The third morning the sun shone and the heavens once more appeared clear. Charles was awake and on the alert long before any of the campers had discovered the change in the weather. Throwing a buffalo robe about him, girding himself with a rope to which hung all the wading shoes the camp could produce, with a kettle on his head and frying pan in hand, which he beat vigorously with an iron spoon, he danced and shouted about every tent, in true Modoc style. Peering out, we inquired of this fantastic "medicine man," the cause for his unearthly din. The only response we had was—"Big Ingin—uh! sun dance—whoop!" when he continued his contortions of body and everlasting clatter until all were aroused to welcome the shining sun. The clouds had all dispersed, the bright, clean foliage glistened, the birds sang merrily, and all nature seemed in tune again. Accordingly, our new camper determined to remain, and, so charmed did he become with camp life, that the next rainy day did not dampen his ardor in the least and at the breaking up, he was the most enthusiastic camper of the party. An unfortunate accident occurred to him later in his camping experience —he lost his diaphragm. A fellow may lose one eye, an arm or leg, without very serious inconvenience, a sacrifice *can* be made of one of these dual organs without endangering life. But the loss of a diaphragm is a very serious affair! He lost it somewhere among the daisies, and, although Charles searched for it faithfully for an entire morning, we at last were compelled to harbor a photographer minus a diaphragm. Writing of daisies reminds us that they grow luxuriously around our camping grounds. A field of fifty acres, near us, is so covered with them as to give it the appearance of being snow clad. As the wind blows over them, inclining all their faces in one direction, the white gives place to the golden yellow, the effect of which, in the bright sunlight, is a charming sight. We made frequent excursions to this field of alternate silver and gold, to watch the ever changing color produced by wind and sun.

One need only quietly observe nature in all her moods to become infatuated with her. There be those who wilfully, or perhaps thoughtlessly, close their eyes and never see anything that is not pointed out to them. To stroll in the woods, or by the meadows with a companion familiar with nature's works, is to be wondrously entertained and surprised too at your own stupidity. "What is that clear, peeping sound I hear?" inquired a friend, as we were riding by a meadow one warm spring morning. Tree frogs, suggested one. "No," said a ruddy-faced country boy. "Them's peeps?" "What are 'peeps?'" we inquired. "Why, they be sunthin' like lizzards. I've watched 'em often and seen 'em make that noise—them's not frogs!" That boy had learned a fact that will stick by him —he had investigated, seen, knew. So with all phenomena not readily understood. Investigate it and you will derive pleasure and knowledge therefrom. We have a camper who clambered over rocks, through briars, and slid down the mountain-side upon his abdomen, riddling clothing and abrading cuticle in an endeavor to obtain a view of that wondrously melodious songster, the wood robin (*turdus melodus*). He failed of his attempt, but learned how and where sundry insects deposited their eggs—that a mosquito emits an electric spark from the point of his abdomen after he has filled his conical little body with your blood; that ants can kill and carry away a caterpillar; that garter snakes will lie in the sun with mouths open while ants and other insects enter therein; that a peculiar abiding place is furnished by nature for the young of a certain insect, through a wound made in a leaf, from which grows a hut with ciliated doors; that a colony of yellow jackets are capable of killing a rattlesnake; that other insects than bees build cells and make honey. These were items of interest and knowledge obtained in a few hours observation upon the mountain side.

When visitors from the city came to us in our mountain retreat, excursions were made to surrounding ravines and other localities of interest and beauty. Frozen Run, a rapid, clear and cold brook, that finds its way over a rocky bed, around boulders—moss-covered and of immense size, forming many pretty falls and sparkling pools—was our favorite picknicking retreat. Ruins of furnaces, railways, dams and old houses, flanked the grass grown road, while the branches of immense beeches and elms completely shut out the sun. Ferns, violets, daisies and mosses flourished underneath, making the old road peculiarly beautiful and attractive. The ladies were carried to the spot—two miles from camp—upon the farm wagon, seated in a row upon a "buck board." The gentlemen carried fishing poles and cameras. Pictures were taken, flowers gathered, and pleasant rambling among the ruins of the picturesque old furnaces, until the lunching hour, when the gentlemen appeared with the highly-colored trout taken from this dark, cold stream, roasted them in beds of glowing coals, made tea, broiled chickens, and spread such a luncheon on a moss-covered rock as would claim the earnest attention of less hungry people.

In the evening we had boat rides upon the pond, accompanied with the melody of the wood thrush; and, later, the shrill and commanding note of the whipporwill. Many pleasant evenings were spent about the camp-fire, special pains always being taken in its construction. Reclining in hammocks, lying upon robes, seated in camp-stools, we arranged ourselves about the bright fire, into which all peered with wonderful attention, watching the glowing coals and building in imagination fantastic figures and weird forms and faces therefrom, all the while relating stories and adventures, but never lifting eyes from the fascinating fire. Hours would be spent in this sort of recreation, until the hooting owls on the mountain would bring us to a realization of the rapidly passing hours, and that Christian bed time had long since glided silently away.

So passed the days and evenings in camp, and in the same manner, and with the same jolly companionship, do we hope to spend many more, in the leafy, flowery Junes to come.

